# Effects of Pretreatment With Coenzyme Q10 (CoQ10) and High‐Intensity Interval Training (HIIT) on FNDC5, Irisin, and BDNF Levels, and Amyloid‐Beta (Aβ) Plaque Formation in the Hippocampus of Aβ‐Induced Alzheimer's Disease Rats

**DOI:** 10.1111/cns.70221

**Published:** 2025-02-17

**Authors:** Samira Puoyan‐Majd, Abdolhossein Parnow, Masome Rashno, Rashid Heidarimoghadam, Alireza komaki

**Affiliations:** ^1^ Bio‐Sciences Department, Physical Education and Sport Sciences Faculty Razi University Kermanshah Iran; ^2^ Neurophysiology Research Center Hamadan University of Medical Sciences Hamadan Iran; ^3^ Asadabad School of Medical Sciences Asadabad Iran; ^4^ Student Research Committee, Asadabad School of Medical Sciences Asadabad Iran; ^5^ Department of Ergonomics School of Health, Hamadan University of Medical Sciences Hamadan Iran; ^6^ Department of Neuroscience School of Science and Advanced Technologies in Medicine, Hamadan University of Medical Sciences Hamadan Iran

**Keywords:** Alzheimer's disease, BDNF, coenzyme Q10, FNDC5, high‐intensity interval training, irisin, rat

## Abstract

**Aims:**

Physical exercise has been shown to protect against cognitive decline in Alzheimer's disease (AD), likely through the upregulation of brain‐derived neurotrophic factor (BDNF). Recent studies have reported that exercise activates the FNDC5/irisin pathway in the hippocampus of mice, triggering a neuroprotective gene program that includes BDNF. This study aimed to investigate the effects of 8 weeks of pretreatment with coenzyme Q10 (CoQ10) and high‐intensity interval training (HIIT), both individually and in combination, on FNDC5, irisin, BDNF, and amyloid‐beta (Aβ) plaque formation in the hippocampus of Aβ‐related AD rats.

**Methods:**

In this study, 72 male Wistar rats were randomly assigned to one of the following groups: control, sham, HIIT (low intensity: 3 min running at 50%–60% VO2max; high intensity: 4 min running at 85%–90% VO2max), Q10 (50 mg/kg, orally administered), Q10 + HIIT, AD, AD + HIIT, AD + Q10, and AD + Q10 + HIIT.

**Results:**

Aβ injection resulted in a trend toward decreased levels of FNDC5, irisin, and BDNF, alongside increased Aβ plaque formation in the hippocampus of Aβ‐induced AD rats. However, pretreatment with CoQ10, HIIT, or their combination significantly restored hippocampal levels of FNDC5, irisin, and BDNF, while also inhibiting Aβ plaque accumulation in these rats.

**Conclusion:**

Pretreatment with CoQ10 and HIIT improved the Aβ‐induced reduction in BDNF levels probably through the FNDC5/irisin pathway and preventing Aβ plaque formation.

## Graphical abstract

This study investigated the effects of 8 weeks of pretreatment with coenzyme Q10 (CoQ10) and high‐intensity interval training (HIIT) alone and in combination on FNDC5, irisin, BDNF, and amyloid‐beta (Aβ) plaque formation in the hippocampus of Aβ‐related AD rats. Pretreatment with CoQ10 and HIIT improved the Aβ‐induced reduction in BDNF levels probably through the FNDC5/irisin pathway and preventing Aβ plaque formation.

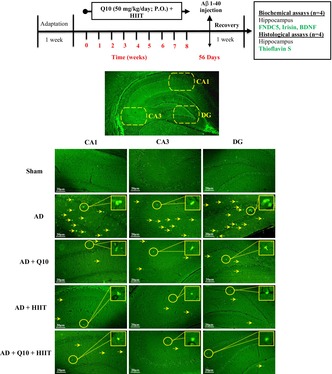

## 1 Introduction

Physical inactivity is the main risk factor for cognitive deficit in Alzheimer's disease (AD) [1]. AD, the commonest form of neurodegenerative dementia, is a progressive neurodegenerative disorder that represents the 7th leading cause of mortality in those aged 65 years [2, 3]. AD is defined by progressive memory and cognitive loss [4], as well as pathophysiological alterations, including extracellular accumulation of insoluble neurofibrillary tangles (NFT) of phosphorylated tau (P‐tau) and β‐amyloid (Aβ) plaques inside the neurons [5, 6]. Aβ generation causes mitochondrial dysfunction, endoplasmic reticulum stress, oxidative stress (OS), or neuroinflammation, consequently neuronal death in AD [5].

Neurotrophin or Brain‐derived neurotrophic factor (BDNF) is involved in the pathogenesis of AD [7, 8]. BDNF belongs to the nerve growth factor family, and is associated with an improvement in learning and memory because it can regulate long‐term depression (LTD) and long‐term potentiation (LTP), proliferation of dendritic arbor, synaptic plasticity, neuronal survival, axonal sprouting, and neuronal differentiation [7, 9, 10]. There are alterations in the BDNF levels in patients with AD [10].

In the early stages of AD, there is an increase in BDNF serum levels, indicating a compensatory repair mechanism in early neurodegeneration and Aβ degradation. As the disease progresses, BDNF is decreased, which is related to the severity of dementia [7]. Several studies have shown that BDNF is induced by exercise in various brain regions, the strongest of which is in the hippocampus [11, 12]. Although the effectiveness of exercise in cognition is widely accepted, the actual mediator of such effect has not yet been identified. Evidence suggests a mechanism in peripheral tissues, such as skeletal muscle and adipose tissue that may respond to exercise by producing leptin and irisin, which enhance brain function [13].

Irisin with 112 amino acids is a novel hormone that mediates the beneficial effects of exercise, by the proteolytic cleavage of fibronectin type III domain‐containing protein 5 (FNDC5) regulated by peroxisome proliferator‐activated receptor‐γ coactivator 1α (PGC1‐α) [12, 13, 14]. Irisin has many functions in the brain, including neurogenesis, neural differentiation, and memory formation by the stimulation of BDNF in the hippocampus [15]. There is an increase in FNDC5/irisin/BDNF levels in the neurons after aerobic exercise [15, 16]. FNDC5 and irisin are the key mediators of the beneficial effects of exercise on memory and synaptic plasticity in AD, thus, the intensity and type of exercise should be considered for the expression of irisin [17]. It is suggested that high‐intensity training compared with low‐intensity training leads to higher irisin and BDNF secretion [18, 19]. High‐intensity interval training (HIIT), in its various forms, is one of the most effective tools for improving brain function today. HIIT consists of multiple bouts of high‐intensity exercise (~85%–95% VO2MAX) lasting 1–4 min with rest or active recovery intervals [20].

Coenzyme Q10 (CoQ10) is the main antioxidant in both mitochondria and lipid membranes and also a strong free radical scavenger. Endogenously, the production of CoQ10 decreases with aging [21]. CoQ10 possesses neuroprotective effects on neurodegenerative diseases, like Parkinson's disease and AD [22]. CoQ10 supplementation reduces OS and Aβ aggregation and improves memory in aged mice [23].

Based on that mentioned issues, we assessed the effect of 8 weeks of HIIT along with CoQ10 supplementation on the prevention of AD‐induced brain damage with emphasis on the FNDC5/irisin/BDNF signaling pathway. Also, thioflavin S staining was used to indicate Aβ plaque generation in the brain tissue.

## 2 Materials and Methods

### 2.1 Animal Study

Seventy‐two adult male Wistar rats (8 weeks; 180 ± 20 g) were provided from the breeding institute of the animal house of Hamadan University of Medical Sciences (UMSHA). They were kept in a room at 22°C ± 2°C under a 12‐h light/dark cycle (light on from 7 a.m. to 7 p.m.) and could drink water and eat food during the experiment. The approval was obtained from the Ethics Committee of the UMSHA (IR.UMSHA.REC.1400.459) and were performed in accordance with the ARRIVE guidelines (Animal Research: Reporting of In Vivo Experiments).

### 2.2 Experimental Design

**FIGURE 1:**
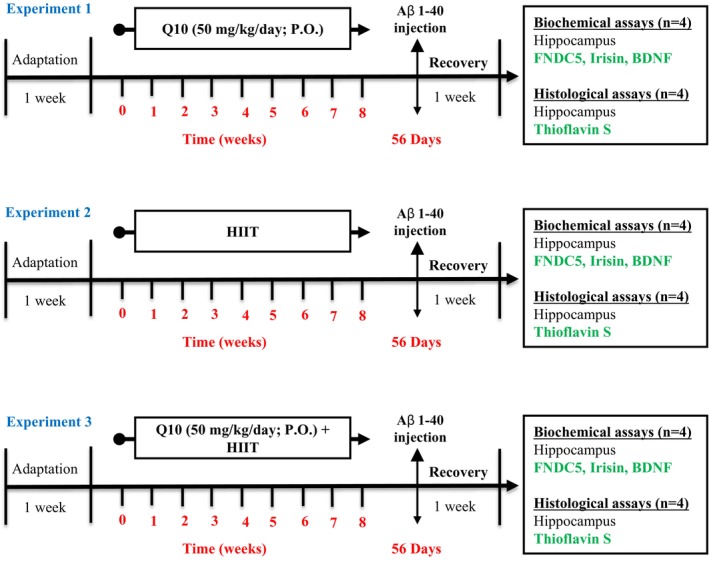
A schematic diagram of the experimental design and treatment schedule. All animals used in this study were divided into three experiments as follows: Experiment 1, after 1 week of adaptation, pre‐treatment of rats with Q10 was initiated and continued for eight consecutive weeks. Then, biochemical (*n* = 4) and histological (*n* = 4) alterations were also examined on the same day. Experiment 2, after 1 week of adaptation, HIIT was performed for eight consecutive weeks. Then, AD was induced by Aβ_1–40_ and after recovery for 1 week. Subsequently, biochemical (*n* = 4) and histological (*n* = 4) alterations were also examined on the same day. Experiment 3, after 1 week of adaptation, HIIT was performed, and also pre‐treatment with Q10 was initiated and continued for eight consecutive weeks. Then, AD was induced by Aβ_1–40_ and after recovery for 1 week. Subsequently, biochemical (*n* = 4) and histological (*n* = 4) alterations were also examined on the same day. Aβ: Amyloid‐beta; HIIT: High‐intensity interval train; BDNF: Brain‐derived neurotrophic factor; HIIT: High‐intensity interval training; FNDC5: Fibronectin type III domain‐containing protein 5; Q10: Coenzyme Q10; P.O.: Per Oral.

Figure 1 displays a schematic of the study design. After acclimatization for a week, animals were randomly allocated to nine groups (*n* = 8): (1) sham: This group received an intracerebroventricular (ICV) injection of phosphate‐buffered saline (PBS) (5 μL/rat), (2) control: healthy rats with free access to water and food, (3) Q10: This group was treated with CoQ10 (50 mg/kg/day; P.O.) for 8 weeks, (4) HIIT: This group performed HIIT on a treadmill for 8 weeks, (5) HIIT + Q10: This group performed HIIT on a treadmill and was treated with CoQ10 (50 mg/kg/day; P.O.) for 8 weeks, (6) AD: This group received Aβ_1–40_ (5 μL/rat, ICV), (7) AD + Q10: This group received CoQ10 (50 mg/kg/day; P.O.) for 8 weeks followed by Aβ_1–40_ (5 μL/rat, ICV), (8) AD + HIIT: This group performed HIIT on a treadmill for 8 weeks, followed by treating with Aβ_1–40_ (5 μL/rat, ICV), and (9) AD + HIIT + Q10: this group performed HIIT on a treadmill followed by treating with CoQ10 (50 mg/kg/day; P.O.) for 8 weeks, and then Aβ_1–40_ (5 μL/rat, ICV).

### 2.3 Preparation and Gavage of CoQ10

The dry powder of CoQ10 supplementation (purchased from Sigma‐Aldrich Co., USA) was dissolved in water to a concentration of 50 mg/cc, then administered orally via gavage using an insulin syringe and gavage needle at a dose of 20 mg/kg. We chose a CoQ10 dose of 50 mg/kg/day, which has been shown to enhance hippocampal synaptic plasticity and cognitive function in a rat model of AD [24, 25]. The treatment duration was set at 8 weeks, as previous studies have demonstrated that this timeframe is sufficient to observe improvements in hippocampal synaptic plasticity and cognitive function in an AD model induced by ICV injection of Aβ_1–40_ [26, 27].

### 2.4 Assessment of Maximal Oxygen Uptake (VO2max) and HIIT Exercise Protocol

HIIT was exercised on a 4‐line treadmill (Tajhiz Gostare Omide Iranian, Iran). After adaptation to the treadmill environment by walking at 5–10 m/min, with a span of 5 min and a slope of 0°, an indirect protocol was applied to assess maximal oxygen uptake (VO2max) as follows: Following 5 min warm‐up, running was performed at 10 m/min for 1 min, followed by increasing the treadmill speed every 3 min at 3 m/min until no possible running by animals [28, 29]. The VO2max speed was the speed at the highest oxygen consumption (or maximal aerobic speed [MAS]) obtained after the exercise period when each rat was not able to maintain the running speed [28, 30]. We determined the maximal running speed at MAS or VO2max and the speed at VO2max before the first and fifth weeks of the program. Afterward, the used intensities were determined according to the considered MAS, which was equal to 100% of VO2max, which occurred at 37 m/min (before the first week) and 43 m/min (before the fifth week). Thus, the HIIT protocol included training sessions consisting of 4 min of running at 85%–90% VO2 max and then 3 min of running at 50%–60% VO2 max, repeated seven times (total: 49 min) (Table 1). We considered 5 min of warm‐up and cool‐down at 40% VO2max before and following the training [30].

**TABLE 1:** HIIT Protocol.

Weeks of training	Warm‐up	Main part of HIIT exercises		Cool‐down	The total exercise time
		High‐intensity interval	Low‐intensity interval		
1–8 weeks	5 min/40% VO2max	28 min (7 Set 4 min)/85%–90% VO2max	21 min (7 Set 3 min)/50%–60% VO2max	5 min/40% VO2max	49 min

*Note:* Slop of the treadmill remains 0°, during the entire steps/stages of the training. To determine the training intensity (VO2max), the exercise capacity test was performed 2 day before the 1st and the 5th week.

### 2.5 AD Induction

Aβ _1–40_ (Tocris Bioscience Co., UK) was injected to create AD, after 8 weeks of HIIT training and receiving CoQ10. In brief, rats were anesthetized intraperitoneally (i.p) with a mixture of ketamine and xylazine (respectively 100 and 10 mg/kg), and located into a stereotaxic device (Stoelting Co., Wood Dale, USA), followed by splitting their scalp. After dissolving Aβ_1–40_ (100 μg) in 100 μL of PBS (vehicle solution), incubation was done at 37°C for 7 days before usage, leading to the development of Aβ plaques toxic to the nervous system [26, 31, 32]. Then, Lambda and Bregma were fixed to the balance level regarding the horizontal plane, followed by drilling a hole over the ventricular area in the skull surface (coordinates: ML: 2 mm lateral, AP: 1.2 mm posterior to the Bregma, and DV: 4 mm below the dura) [33]. A 5 μL microsyringe (Hamilton Laboratory Products, USA) was employed for the injections and then, the scalp skin was sutured and 1 week of recovery was considered in a warm cage [34].

### 2.6 Biochemical Assay

At the final stage of the experiments, animals were deeply anesthetized with a ketamine‐xylazine mixture (100 mg/kg and 10 mg/kg, respectively). After euthanasia, the brains were removed (*n* = 4 per group), and the hippocampal tissue was immediately dissected on ice and washed with saline. The specimens were kept at −80°C until tissue processing. Real‐time quantitative PCR (qPCR) analyzed the FNDC5 expression levels. Then, the reaction mixture (20 μL), qPCR Master (10 μL), forward primer (1 μL), reverse primer (1 μL), template DNA (1 μL), and RNase‐free (up to 20 μL) were added to each sample in a clean microtube and mixed by pipetting or vortexing. In‐house‐designed primers were applied to quantify glyceraldehyde 3‐phosphate dehydrogenase gene (GAPDH). Sequences and annealing temperatures were as follows: GAPDH Fwd: 5′ GAG CCT TCG ACC ACT GCT AC 3′, temperature: 60°C; GAPDH Rev.: 5′ ATT TCA TCC AGG CTG TCC AC 3′, temperature: 60°C. Each specimen was analyzed two times. The PCR cycle conditions were as follows: +95°C for 5 min and 40 cycles at +95°C and at +60°C for 30s. FNDC5 relative expression levels were determined using the ΔΔ*Ct* method and normalized to the GAPDH expression.

Hippocampal BDNF and irisin levels were assessed by a rat irisin ELISA kit (ZellBio, Germany Cat. No. ZB‐1765‐R9648) and rat BDNF ELISA kit (ZellBio, Germany Cat. No. 0476‐R9648) as instructed.

### 2.7 Thioflavin S Staining Protocol

**FIGURE 2:**
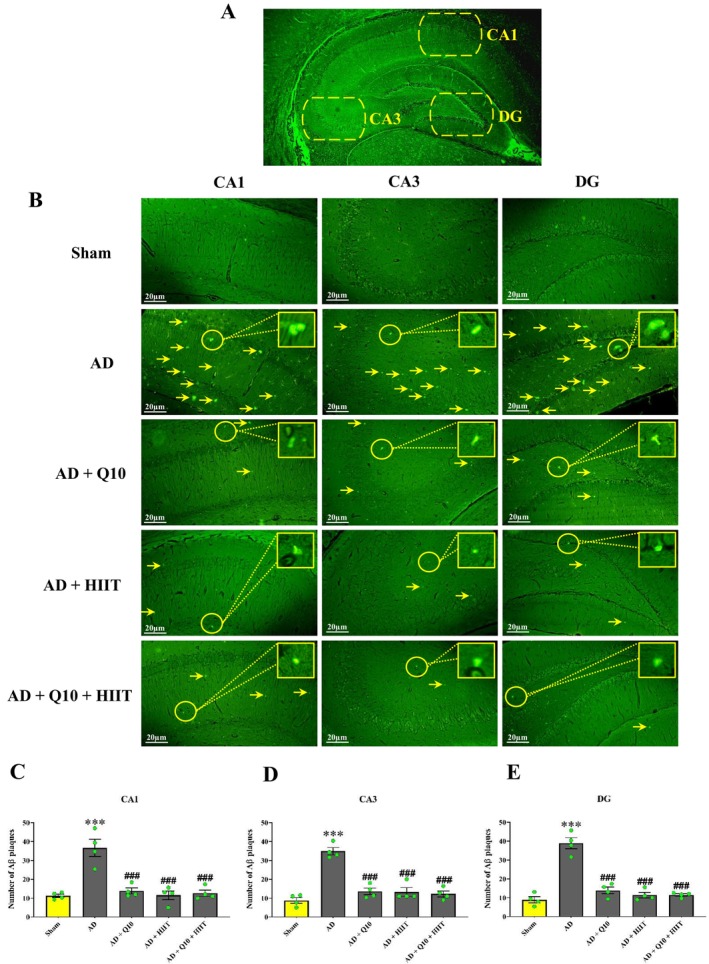
Effects of 8 weeks of treatment with Q10 (50 mg/kg/day; P.O.) and HIIT on extracellular Aβ plaque formation using thioflavin S staining in the hippocampus of Aβ‐exposed rats. “A” shows a photomicrograph of the hippocampal CA1, CA3, and DG areas of rats. “B” represents the revealing photographs of extracellular Aβ plaques (Aβ plaques are shiny green, recognized by yellow arrows; 100 × magnification, scale bar 20 μm). The quantitative data of the number of Aβ plaques are shown in the hippocampal CA1 (C), CA3 (D), and DG (E) regains. The values represent the count of Aβ plaques per 0.01 mm^2^, which were obtained from two or three fields of each sample (*n* = 4; mean ± SEM). Sham group: This group received an intracerebroventricular (ICV) injection of phosphate‐buffered saline (PBS) (5 μL/rat), control group: Healthy rats with free access to water and food, Q10 group: This group was treated with CoQ10 (50 mg/kg/day; P.O.) for 8 weeks, HIIT group: This group performed HIIT on a treadmill for 8 weeks, HIIT + Q10 group: This group performed HIIT on a treadmill and was treated with CoQ10 (50 mg/kg/day; P.O.) for 8 weeks, AD group: This group received Aβ_1–40_ (5 μL/rat, ICV), AD + Q10 group: This group received CoQ10 (50 mg/kg/day; P.O.) for 8 weeks followed by Aβ_1–40_ (5 μL/rat, ICV), AD + HIIT group: This group performed HIIT on a treadmill for 8 weeks, followed by treating with Aβ_1–40_ (5 μL/rat, ICV), and AD + HIIT + Q10 group: This group performed HIIT on a treadmill followed by treating with CoQ10 (50 mg/kg/day; P.O.) for 8 weeks, and then Aβ_1–40_ (5 μL/rat, ICV). ***p < 0.001 vs. the sham group; ###*p* < 0.001 vs. the AD group (one‐way ANOVA; Tukey's post hoc test). AD: Alzheimer's disease; Aβ: Amyloid‐beta; CA1: Cornu ammonis 1; CA3: Cornu ammonis 3; DG: Dentate gyrus; HIIT: High‐intensity interval training; Q10: Coenzyme Q10.

At the end of the experiment, animals were anesthetized via intraperitoneal (i.p.) injection of ketamine (100 mg/kg) and xylazine (10 mg/kg). Following anesthesia, samples were perfused intracardially with ice‐cold 5% formaldehyde. Aβ plaque formation in the CA1, CA3, and DG regions of the hippocampus was assessed using thioflavin S staining (Figure 2A). The brain was removed and fixed in formalin 10, and then, they were dehydrated, embedded in paraffin, and cut into consecutive 6‐μm transverse sections. Then, staining using 1% Thioflavin‐S was done in a dark environment for 10 min [35]. Using distilled water and 70% ethanol, the slides were washed and finally, studied by a fluorescent microscope. An average of two or three slides were reported for each animal.

### 2.8 Statistical Analysis

GraphPad Prism 5.0 was used for graph construction and data analysis. The homogeneity of variance was assessed using Levene's test, and normality was evaluated with a quantile‐quantile (QQ) plot. FNDC5 levels were analyzed using the Kruskal–Wallis *H* test (ANOVA on ranks), followed by Dunn's test to compare median values among groups with non‐normally distributed data. A box plot was used to visualize the nonparametric data. For other numerical data, a parametric one‐way analysis of variance (ANOVA) followed by Tukey's test was performed, and results were expressed as the mean ± standard error of the mean (SEM). *p* values < 0.05 were considered statistically significant.

## 3 Results

### 3.1 Q10 and HIIT Improved Hippocampal Neurotrophic Factors in AD Rats

**FIGURE 3:**
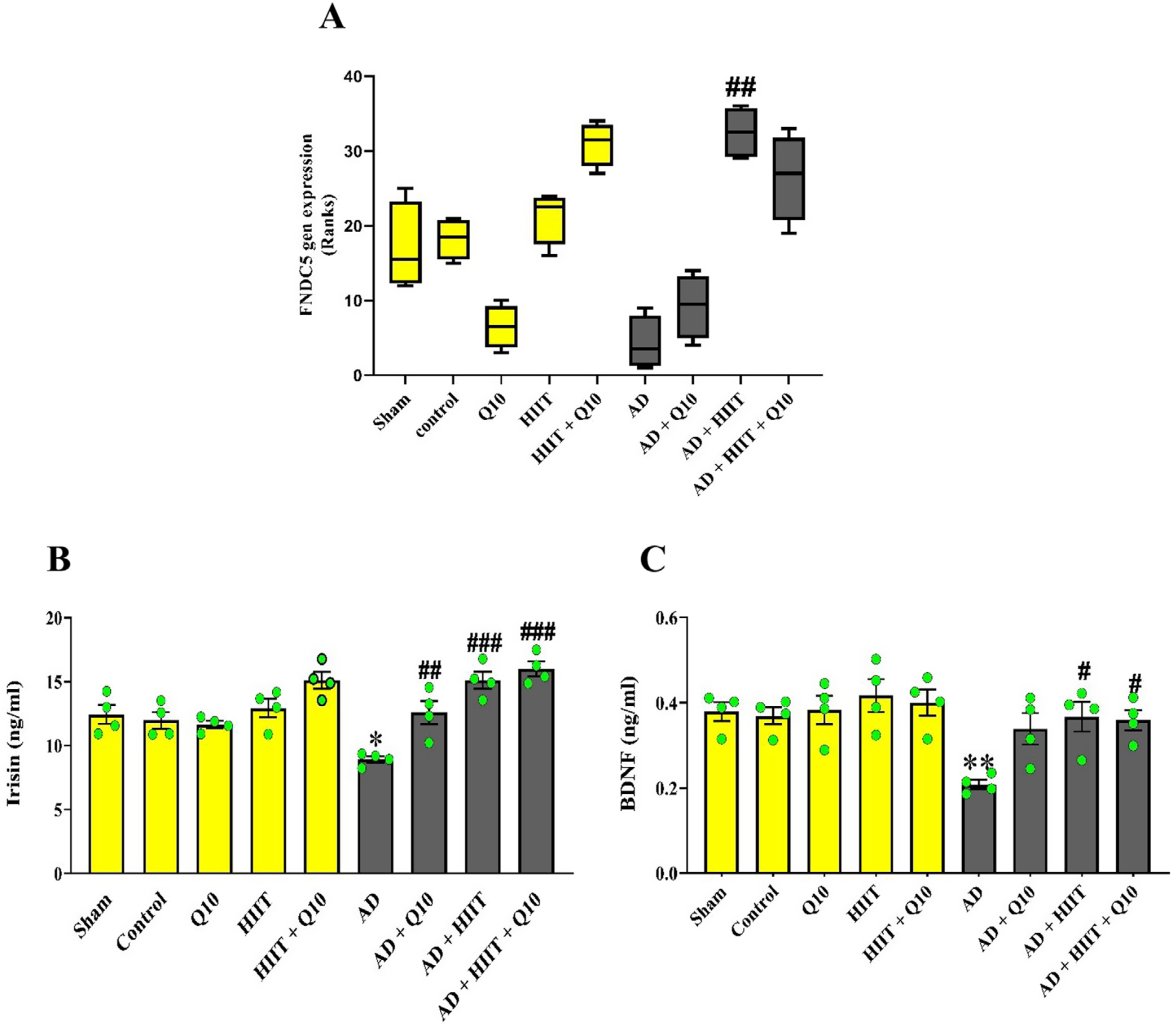
Effects of 8 weeks of treatment with Q10 (50 mg/kg/day; P.O.) and HIIT on neurotrophic factors in the hippocampus of Aβ‐exposed rats. FNDC5 expression was evaluated using RT‐PCR (A). Irisin (B) and BDNF (C) levels were assessed using ELISA kits (*n* = 4; mean ± SEM). Sham group: This group received an intracerebroventricular (ICV) injection of phosphate‐buffered saline (PBS) (5 μL/rat), control group: healthy rats with free access to water and food, Q10 group: This group was treated with CoQ10 (50 mg/kg/day; P.O.) for 8 weeks, HIIT group: This group performed HIIT on a treadmill for 8 weeks, HIIT + Q10 group: This group performed HIIT on a treadmill and was treated with CoQ10 (50 mg/kg/day; P.O.) for 8 weeks, AD group: This group received Aβ_1–40_ (5 μL/rat, ICV), AD + Q10 group: This group received CoQ10 (50 mg/kg/day; P.O.) for 8 weeks followed by Aβ_1–40_ (5 μL/rat, ICV), AD + HIIT group: This group performed HIIT on a treadmill for 8 weeks, followed by treating with Aβ_1–40_ (5 μL/rat, ICV), and AD + HIIT + Q10 group: This group performed HIIT on a treadmill followed by treating with CoQ10 (50 mg/kg/day; P.O.) for 8 weeks, and then Aβ_1–40_ (5 μL/rat, ICV). *p < 0.05 and **p < 0.01 vs. the sham group; #*p* < 0.05, ##*p* < 0.01, and ###*p* < 0.001 vs. the AD group (one‐way ANOVA; Tukey's post hoc test). AD: Alzheimer's disease; Aβ: Amyloid‐beta; BDNF: Brain‐derived neurotrophic factor; HIIT: High‐intensity interval training; FNDC5: Fibronectin type III domain‐containing protein 5; RT‐PCR: Real‐time PCR; Q10: Coenzyme Q10.

As shown in Figure 3A, decreasing neuronal FNDC5 gene expression was observed in the AD group compared to sham group, although its trend was not statistically different (*F*_8,27_ = 0.9705, *p* > 0.05, Figure 3A). Notably, pretreatment with HIIT caused increasing FNDC5 gene expression in the AD + HIIT group compared to the AD group (*p* < 0.01).

As shown in Figure 3B, a trend toward increasing irisin levels was observed in the HIIT + Q10 group compared to normal rats. Irisin levels in the AD group significantly reduced compared to the sham group (*F*_8,27_ = 11.64, *p* < 0.05). Notably, Q10 and HIIT increased irisin levels in the AD + Q10, AD + HIIT, and AD + HIIT + Q10 groups than in the AD group (*p* < 0.01, *p* < 0.001, and *p* < 0.001, respectively).

BDNF levels significantly decreased following Aβ injection in the hippocampus of the AD group compared to the sham group (*F*_8,27_ = 11.64, *p* < 0.01). However, HIIT and HIIT + Q10 increased BDNF levels in both the AD + HIIT + Q10 and AD + HIIT groups than in the AD group (*p* < 0.05 for each, Figure 3C).

### 3.2 Q10 and HIIT Prevent Aβ Plaque Formation in the Hippocampus of AD Rats

Aβ plaque generation was observed in the hippocampus using thioflavin S staining (Figure 2A,B). The control groups (sham, HIIT, Q10, control, and HIIT + Q10) showed low levels of Aβ deposition in the hippocampal CA1, CA3, and DG regions and did not differ from each other (*p* > 0.05, data not shown). However, Aβ plaque formation was detected in the hippocampal CA1 (*F*_4,15_ = 18.88; *p* < 0.001, Figure 2C), CA3 (*F*_4,15_ = 32.89; *p* < 0.001, Figure 2D), and DG (*F*_4,15_ = 46.44; *p* < 0.001, Figure 2E) regions of the AD group than in the sham group. The number of Aβ plaques significantly decreased in the hippocampal CA1, CA3, and DG regions in the AD + HIIT, AD + Q10, and AD + Q10 + HIIT groups in comparison with the AD group (*p* < 0.001 for all).

## 4 Discussion

AD is a progressive neurodegenerative disease characterized by the accumulation of Aβ in the spaces between neuronal cells, known as plaques, which disrupt the neuron signaling pathway, and NFTs, which are intracellular protein tangles. Currently, the Aβ hypothesis is widely accepted. Accordingly, we used Aβ_1–40_ injection into the hippocampus to induce an AD model. ICV injection of Aβ caused the following events: (1) decreased FNDC5 gene expression in the hippocampus of AD rats, (2) decreased irisin and BDNF levels in the hippocampus of AD rats, and (3) increase Aβ plaques in the hippocampal CA1, CA3, and DG areas in AD rats. In contrast, pretreatment with HIIT and CoQ10 for eight consecutive weeks ameliorated FNDC5 expression and irisin and BDNF levels and decreased accumulation of the Aβ plaques in the hippocampal CA1, CA3, and DG regions in AD rats.

BDNF belongs to the neurotrophin family of growth factors and is associated with hippocampal function, synaptic plasticity, and learning through the interaction with its high‐affinity receptor Trk B [36]. BDNF can promote several aspects of brain development, such as differentiation, neuronal viability, migration, synaptogenesis, and dendritic arborization [11]. In addition, BDNF is essential for synaptic plasticity, hippocampal function, and learning [12]. There is an association between BDNF and AD pathogenesis [37]. Both hippocampal BDNF mRNA and protein levels are significantly reduced in post‐mortem brain areas of AD patients [33, 38, 39]. An in vitro study also reported a decrease in BDNF transcript following exposure to oligomeric Aβ [40, 41]. In the present work, decreased BDNF levels were found in the hippocampal tissues of the Aβ‐induced AD rats. The following molecular mechanisms can significantly decrease BDNF levels in AD: (1) The Aβ aggregation can directly suppress the proteolytic conversion of BDNF from pro‐BDNF and reduce its concentrations [42]; (2) the Aβ oligomers lead to a deficit in BDNF‐facilitated TrkB retreated handling through the interruption in ubiquitin and calcium homeostasis [43, 44]; (3) Aβ decreases phosphorylated cAMP‐responsive element‐binding protein (CREB), which regulates BDNF transcript expression [43, 44]; (4) Aβ activity leads to the calpain overproduction in AD, and then calpain induces cleavage in TrkB‐FL receptors, and reduces their levels [45]; (5) increased glucocorticoids in AD and continuous exposure to glucocorticoids, such as corticosterone reduced BDNF expression at both the protein and mRNA levels [43]; (6) Increased OS is associated with reduced BDNF levels [46], and OS plays a crucial role in the development and progression of cognitive impairment in [47]. OS arises from excessive reactive oxygen species (ROS) levels [48], and exposure to Aβ leads to ROS generation [49, 50]. Consistent with these findings, our previous study demonstrated that ICV injection of Aβ_1–40_ decreased the activities of total thiol groups (TTG), catalase (CAT), and glutathione peroxidase (GPx), all of which are endogenous antioxidants, whereas the level of malondialdehyde (MDA), an indicator of lipid peroxidation, increased in the hippocampus [51]. Several mechanisms have been proposed through which OS could reduce BDNF levels, including decreased CREB activity, increased NF‐κB DNA‐binding, and energy depletion [46]; and (7) finally, mitochondrial dysfunction due to Aβ as a main feature of AD is linked to differences in axonal transport of BDNF [44]. The effectiveness of pretreatment on BDNF in the HIIT, Q10, and Q10 + HIIT groups was approved by an increase in its levels in the hippocampus, which is consistent with earlier reports [52]. The mechanisms underlying how exercise‐related increases cerebral BDNF levels are still being studied, and currently, three distinct pathways emerge as key contributors [53]. The first pathway involves the hemodynamic response to exercise, in which an increase in shear stress on endothelial cells lining blood vessels leads to the NO‐dependent synthesis of BDNF [54]. The second pathway is the neuronal pathway that via calcium influx and neurotransmitter release leading to increased BDNF levels [36]. Finally, the third pathway recently identified involves the secretion into the bloodstream of myokines such as irisin by skeletal muscles during contraction, potentially influencing brain [37, 53, 55, 56]. Irisin is the upstream mediator of BDNF production and triggers the expression of BDNF [11, 57]. Irisin is the cleavage product of FNDC5, produced by adipose tissue, muscle tissue, and the cerebral hippocampus following exercise [58, 59]. FNDC5 has been shown to be regulated by the transcriptional PGC‐1α in skeletal muscle and hippocampal neurons in vivo and in vitro [11, 14, 60]. Exercise can increase PGC1‐α expression mainly in the skeletal muscles and improve different metabolic factors, such as PGC1‐α phosphorylation, AMPK activation, FNDC5 production, and insulin signaling and sensitivity, and consequently the cleavage in FNDC5 to secrete irisin [61]. According to in vitro studies, there is a relationship between irisin and its progenitor FNDC5 and neuroplasticity and BDNF in the central nervous system, as part of a pathway between cognition and physical activity [11, 62]. FNDC5 expression is induced by exercise in the hippocampus in mice, which in turn, can activate BDNF and other neuroprotective genes [56, 60]. Moreover, peripheral delivery of FNDC5 to the liver via adenoviral vectors, resulting in elevated blood irisin, induced expression of BDNF and other neuroprotective genes in the hippocampus. These data indicate that either irisin itself can cross the blood–brain‐barrier (BBB) to induce these gene expression changes or irisin induces a factor x that can [12]. Thereby, irisin could serve as a potential mediator between muscle and brain in pathophysiological conditions. In addition to its peripheral expression, FNDC5/irisin is also produced in different brain regions [53]. In agreement with previous studies, it has been shown that irisin is observed in the cerebrospinal fluid (CSF), purkinje cells of the cerebellum [63], hippocampus, and hypothalamus [11, 64], and FNDC5 is known to be highly expressed in glia (e.g., astrocytes and microglia) and neurons in various brain regions [65]. The irisin‐BDNF axis can strengthen memory function [56] as irisin can enhance BDNF synthesis, glucose homeostasis, hippocampal neurogenesis, and synaptic plasticity, and decrease OS [58, 66]. Lourenco et al. [17] showed a decrease in FNDC5/irisin levels in the human AD brains and cerebrospinal fluid and also in mice with AD. They reported that increased brain or peripheral FNDC5/irisin concentrations attenuate memory and synaptic impairments in AD mouse models. Wrann et al. [11] reported that exercise increases cerebral BDNF expression through the PGC‐1α/FNDC5 pathway, whereas the knockdown of PGC‐1α reduced FNDC5 expression in the brain. Previous studies have shown hippocampal BDNF expression was dependent on exercise intensity [53]. Afzalpour et al. [67] reported that hippocampal BDNF expression and neurogenesis were higher in rats subjected to 4 weeks of high‐intensity interval training (HIIT) compared moderate‐intensity continuous training (MICT) (30 min/day, 5 days/week). Interestingly, previous studies have shown that serum irisin levels followed the same variation pattern as hippocampal BDNF expression in response to exercise [53]. Indeed, it has already been reported that serum irisin levels were higher after HIIT than MICT with five sessions/week for 8 weeks [68] or with five sessions/week for 6 weeks [69]. On the other hand, CoQ10 elevated the BDNF levels in the brain and enhanced the BDNF signaling pathway, indicating the CoQ10 neuroprotective effects [70]. Sheykhhasan et al. [71] showed that CoQ10 administration could increase BDNF levels and neurogenesis in the hippocampus of AD rats. Consistent with our results, irisin levels were reported with an association with BDNF and cognition, particularly hippocampus‐associated memory functions Irisin is regulated by exercise [17, 61]. Youssef et al. [72] showed that exercise (5 days per week for 6 weeks) significantly improved FNDC5 gene expression and irisin levels in diabetic rats but combined quercetin and CoQ10 supplements with exercise significantly increased their levels than exercise alone. Consistent with previous studies, our results demonstrated a decrease in FNDC5 gene expression and irisin protein levels in the hippocampus of Aβ‐induced AD rats. However, pretreatment with HIIT and CoQ10 led to increased FNDC5 expression and irisin levels in the hippocampus, which were associated with elevated BDNF levels. This relationship between FNDC5, irisin, BDNF, and cognition suggests that BDNF may act as a mediator linking cognition and irisin. In line with our findings, previous studies have reported a correlation between irisin and BDNF levels, as well as hippocampal cognitive and memory functions in response to exercise [17, 61].

The accumulation of Aβ in the brain is thought to be a primary clinical and main pathological hallmark of AD [73]. In the present study, according to thioflavin S staining, Aβ injection into the brain increased Aβ plaque formation in the hippocampal CA1, CA3, and DG areas, wherea pretreatment with Q10, HIIT, and Q10 + HIIT in Aβ‐induced AD rats reduced the Aβ plaque formation. These results are in line with the findings of Zhang et al. [73] who reported that treadmill exercise significantly decreased the number of Aβ plaques and the Aβ protein levels. Another study demonstrated that voluntary exercise promotes Aβ clearance and reduces the activation of astrocytes and microglia in aged mice [74]. Consistent with these findings, several studies have also shown that exercise significantly decreases the number of Aβ plaques and Aβ protein levels [73, 75, 76, 77, 78, 79, 80, 81]. Despite the accumulating reports on exercise intervention, the precise mechanism of its beneficial effect remains largely unknown. Recently, reported that overexpression of FNDC5/irisin reversed the suppressive effect of Aβ on BDNF, preventing neuronal apoptosis [59, 63]. The APP/FNDC5 interaction, which occurs when FNDC5 binds to a specific domain between the β‐ and α‐secretase cleavage sites of APP, can reduce Aβ formation and lead to a decrease in Aβ surface secretion in the culture medium [82]. Importantly, it has been suggested that FNDC5 may inhibit the cleavage of β‐secretase by binding to the N‐terminus of the C99 fragment [63]. CoQ10 also reduced Aβ plaque formation and improved behavioral deficits in AD rat [24, 83, 84, 85, 86]. However, further research is needed to fully elucidate the role of exercise and CoQ10 in the nervous system, particularly in the prevention of AD.

## 5 Conclusion

**FIGURE 4:**
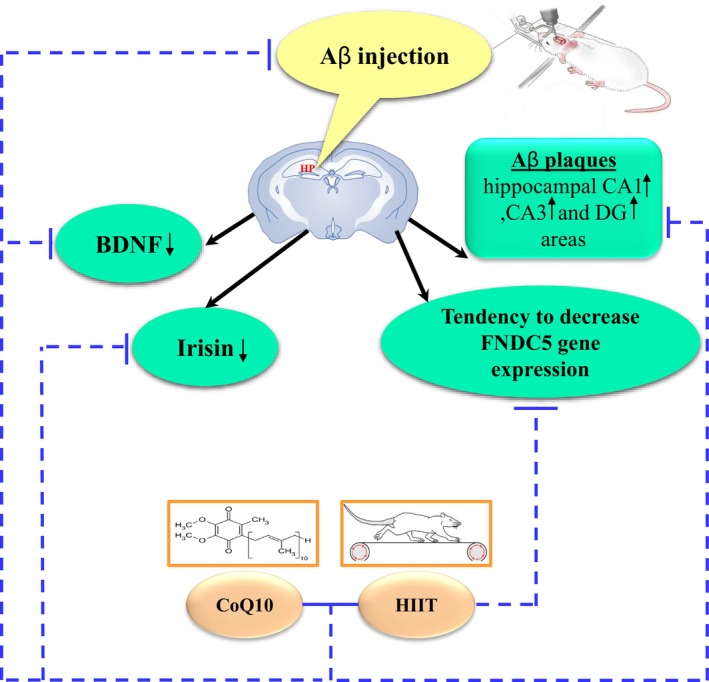
Schematic representation of the “Protective Effects of CoQ10 and HIIT in Aβ‐induced AD Rats: Possible Involved Mechanisms.” HP hippocampus; BDNF: Brain‐derived neurotrophic factor; FNDC5: Fibronectin type III domain‐containing protein 5; Aβ: Amyloid‐beta; HIIT: High Intensity Interval Training.

In summary, pretreatment with CoQ10 and HIIT improves Aβ‐induced decrease in BDNF probably through the FNDC5/irisin pathway. Therefore, a combination of HIIT and CoQ10 can be a potential factor in preventing Aβ plaque formation and cognitive deficits (Figure 4).

## Author Contributions

S.P.‐M.: experimental work, figure preparation, data analysis, and manuscript drafting. A.P. and M.R.: experimental work, data analysis, and figure preparation. R.H.: experimental work and data analysis. A.K.: conceived the idea, experimental design, funding, data analysis, figure preparation, and manuscript drafting and editing. All authors approved the submitted version.

## Ethics Statement

All of the experiments and animal care methods were confirmed by the Veterinary Ethics Board of the Hamadan University of Medical Science (IR.UMSHA.REC.1400.459) and carried out in accordance with the ARRIVE guidelines (Animal Research: Reporting of In Vivo Experiments).

## Conflicts of Interest

The authors declare no conflicts of interest.

## Data Availability

The data that support the findings of this study are available from the corresponding author upon reasonable request.

## References

[1] E. Duzel , H. van Praag , and M. Sendtner , “Can Physical Exercise in Old Age Improve Memory and Hippocampal Function?,” Brain 139, no. 3 (2016): 662–673. 26912638 10.1093/brain/awv407 PMC4766381

[2] A. A. Tahami Monfared , W. Ye , A. Sardesai , et al., “A Path to Improved Alzheimer's Care: Simulating Long‐Term Health Outcomes of Lecanemab in Early Alzheimer's Disease From the CLARITY AD Trial,” Neurology and Therapy 12 (2023): 1–19. 10.1007/s40120-023-00473-w PMC10195966 37009976

[3] S. López‐Ortiz , J. Pinto‐Fraga , P. L. Valenzuela , et al., “Physical Exercise and Alzheimer's Disease: Effects on Pathophysiological Molecular Pathways of the Disease,” International Journal of Molecular Sciences 22, no. 6 (2021): 2897. 33809300 10.3390/ijms22062897 PMC7999827

[4] G.‐Y. Choi , H.‐B. Kim , E.‐S. Hwang , et al., “Naringin Enhances Long‐Term Potentiation and Recovers Learning and Memory Deficits of Amyloid‐Beta Induced Alzheimer's Disease‐Like Behavioral Rat Model,” Neurotoxicology 95 (2023): 35–45. 36549596 10.1016/j.neuro.2022.12.007

[5] Z.‐X. Tan , F. Dong , L.‐Y. Wu , Y.‐S. Feng , and F. Zhang , “The Beneficial Role of Exercise on Treating Alzheimer's Disease by Inhibiting β‐Amyloid Peptide,” Molecular Neurobiology 58, no. 11 (2021): 5890–5906. 34415486 10.1007/s12035-021-02514-7

[6] S. Takeda , N. Sato , and R. Morishita , “Systemic Inflammation, Blood‐Brain Barrier Vulnerability and Cognitive/Non‐Cognitive Symptoms in Alzheimer Disease: Relevance to Pathogenesis and Therapy,” Frontiers in Aging Neuroscience 6 (2014): 171. 25120476 10.3389/fnagi.2014.00171 PMC4114193

[7] C. Laske , E. Stransky , T. Leyhe , et al., “Stage‐Dependent BDNF Serum Concentrations in Alzheimer's Disease,” Journal of Neural Transmission 113 (2006): 1217–1224. 16362629 10.1007/s00702-005-0397-y

[8] T. Aarons , S. Bradburn , A. Robinson , A. Payton , N. Pendleton , and C. Murgatroyd , “Dysregulation of BDNF in Prefrontal Cortex in Alzheimer's Disease,” Journal of Alzheimer's Disease 69, no. 4 (2019): 1089–1097. 10.3233/JAD-190049 31127785

[9] T. Leyhe , G. W. Eschweiler , E. Stransky , et al., “Increase of BDNF Serum Concentration in Lithium Treated Patients With Early Alzheimer's Disease,” Journal of Alzheimer's Disease 16, no. 3 (2009): 649–656. 10.3233/JAD-2009-1004 19276559

[10] J. Budni , T. Bellettini‐Santos , F. Mina , M. L. Garcez , and A. I. Zugno , “The Involvement of BDNF, NGF and GDNF in Aging and Alzheimer's Disease,” Aging and Disease 6, no. 5 (2015): 331–341. 26425388 10.14336/AD.2015.0825 PMC4567216

[11] C. D. Wrann , J. P. White , J. Salogiannnis , et al., “Exercise Induces Hippocampal BDNF Through a PGC‐1α/FNDC5 Pathway,” Cell Metabolism 18, no. 5 (2013): 649–659. 24120943 10.1016/j.cmet.2013.09.008 PMC3980968

[12] C. D. Wrann , “FNDC5/Irisin–Their Role in the Nervous System and as a Mediator for Beneficial Effects of Exercise on the Brain,” Brain Plasticity 1, no. 1 (2015): 55–61. 28480165 10.3233/BPL-150019 PMC5419585

[13] J. Zhang and W. Zhang , “Can Irisin Be a Linker Between Physical Activity and Brain Function?,” Biomolecular Concepts 7, no. 4 (2016): 253–258. 27356237 10.1515/bmc-2016-0012

[14] P. Boström , J. Wu , M. P. Jedrychowski , et al., “A PGC1‐α‐Dependent Myokine That Drives Brown‐Fat‐Like Development of White Fat and Thermogenesis,” Nature 481, no. 7382 (2012): 463–468. 22237023 10.1038/nature10777 PMC3522098

[15] O. K. Fuller , M. Whitham , S. Mathivanan , and M. A. Febbraio , “The Protective Effect of Exercise in Neurodegenerative Diseases: The Potential Role of Extracellular Vesicles,” Cells 9, no. 10 (2020): 2182. 32998245 10.3390/cells9102182 PMC7599526

[16] G. C. Gonçalves1 , L. H. Venturelli , L. M. C. Sarmento , et al., “The Relationship of Irisin Hormone Released During Physical Exercise and Alzheimer's Disease: A Literature Review,” Revolution Medicines 102 (2023): 194527.

[17] M. V. Lourenco , R. L. Frozza , G. B. de Freitas , et al., “Exercise‐Linked FNDC5/Irisin Rescues Synaptic Plasticity and Memory Defects in Alzheimer's Models,” Nature Medicine 25, no. 1 (2019): 165–175. 10.1038/s41591-018-0275-4 PMC6327967 30617325

[18] H. Quan , E. Koltai , K. Suzuki , et al., “Exercise, Redox System and Neurodegenerative Diseases,” Biochimica et Biophysica Acta (BBA) ‐ Molecular Basis of Disease 1866, no. 10 (2020): 165778. 32222542 10.1016/j.bbadis.2020.165778

[19] Y. Tsuchiya , D. Ando , K. Goto , M. Kiuchi , M. Yamakita , and K. Koyama , “High‐Intensity Exercise Causes Greater Irisin Response Compared With Low‐Intensity Exercise Under Similar Energy Consumption,” Te Tohoku Journal of Experimental Medicine 233, no. 2 (2014): 135–140. 10.1620/tjem.233.135 24910199

[20] E. G. Ciolac , “High‐Intensity Interval Training and Hypertension: Maximizing the Benefits of Exercise?,” American Journal of Cardiovascular Disease 2, no. 2 (2012): 102–110. 22720199 PMC3371620

[21] M. F. Beal , “Mitochondrial Dysfunction and Oxidative Damage in Alzheimer's and Parkinson's Diseases and Coenzyme Q 10 as a Potential Treatment,” Journal of Bioenergetics and Biomembranes 36 (2004): 381–386. 15377876 10.1023/B:JOBB.0000041772.74810.92

[22] M. Spindler , M. F. Beal , and C. Henchcliffe , “Coenzyme Q10 Effects in Neurodegenerative Disease,” Neuropsychiatric Disease and Treatment 5 (2009): 597–610. 19966907 10.2147/ndt.s5212 PMC2785862

[23] G. Omidi , S. A. Karimi , A. Rezvani‐Kamran , A. Monsef , S. Shahidi , and A. Komaki , “Effect of Coenzyme Q10 Supplementation on Diabetes Induced Memory Deficits in Rats,” Metabolic Brain Disease 34 (2019): 833–840. 30848472 10.1007/s11011-019-00402-7

[24] H. Komaki , N. Faraji , A. Komaki , et al., “Investigation of Protective Effects of Coenzyme Q10 on Impaired Synaptic Plasticity in a Male Rat Model of Alzheimer's Disease,” Brain Research Bulletin 147 (2019): 14–21. 30721766 10.1016/j.brainresbull.2019.01.025

[25] M. Asadbegi , H. Komaki , N. Faraji , et al., “Effectiveness of Coenzyme Q10 on Learning and Memory and Synaptic Plasticity Impairment in an Aged Aβ‐Induced Rat Model of Alzheimer's Disease: A Behavioral, Biochemical, and Electrophysiological Study,” Psychopharmacology 240, no. 4 (2023): 951–967. 36811650 10.1007/s00213-023-06338-2

[26] S. Safari , N. Mirazi , N. Ahmadi , et al., “The Protective Effects of Policosanol on Learning and Memory Impairments in a Male Rat Model of Alzheimer's Disease,” Molecular Neurobiology 60, no. 5 (2023): 2507–2519. 36680733 10.1007/s12035-023-03225-x

[27] S. Safari , N. Mirazi , N. Ahmadi , et al., “Policosanol Protects Against Alzheimer's Disease‐Associated Spatial Cognitive Decline in Male Rats: Possible Involved Mechanisms,” Psychopharmacology 240, no. 4 (2023): 755–767. 36723631 10.1007/s00213-023-06317-7

[28] J. C. Ferreira , N. P. Rolim , J. B. Bartholomeu , C. A. Gobatto , E. Kokubun , and P. C. Brum , “Maximal Lactate Steady State in Running Mice: Effect of Exercise Training,” Clinical and Experimental Pharmacology and Physiology 34, no. 8 (2007): 760–765. 17600553 10.1111/j.1440-1681.2007.04635.x

[29] P. Gholipour , A. Komaki , H. Parsa , and M. Ramezani , “Therapeutic Effects of High‐Intensity Interval Training Exercise Alone and Its Combination With Ecdysterone Against Amyloid Beta‐Induced Rat Model of Alzheimer's Disease: A Behavioral, Biochemical, and Histological Study,” Neurochemical Research 47, no. 7 (2022): 2090–2108. 35484426 10.1007/s11064-022-03603-2

[30] K. Lu , L. Wang , C. Wang , Y. Yang , D. Hu , and R. Ding , “Effects of High‐Intensity Interval Versus Continuous Moderate‐Intensity Aerobic Exercise on Apoptosis, Oxidative Stress and Metabolism of the Infarcted Myocardium in a Rat Model,” Molecular Medicine Reports 12, no. 2 (2015): 2374–2382. 25936391 10.3892/mmr.2015.3669

[31] L. Li , J. Dai , L. Ru , G. Yin , and B. Zhao , “Effects of Tanshinone on Neuropathological Changes Induced by Amyloid Beta‐Peptide (1−40) Injection in Rat Hippocampus,” Acta Pharmacologica Sinica 25 (2004): 861–868. 15210058

[32] A. Xuan , D. Long , J. Li , et al., “Hydrogen Sulfide Attenuates Spatial Memory Impairment and Hippocampal Neuroinflammation in Beta‐Amyloid Rat Model of Alzheimer's Disease,” Journal of Neuroinflammation 9, no. 1 (2012): 1–11. 22898621 10.1186/1742-2094-9-202 PMC3458984

[33] Paxinos G. , and C. Watson , The rat brain in stereotaxic coordinates: hard cover edition, (Elsevier, 2006).

[34] M. Asadbegi , P. Yaghmaei , I. Salehi , A. Komaki , and A. Ebrahim‐Habibi , “Investigation of Thymol Effect on Learning and Memory Impairment Induced by Intrahippocampal Injection of Amyloid Beta Peptide in High Fat Diet‐Fed Rats,” Metabolic Brain Disease 32, no. 3 (2017): 827–839. 28255862 10.1007/s11011-017-9960-0

[35] B. Lochocki , T. H. Morrema , F. Ariese , J. J. Hoozemans , and J. F. de Boer , “The Search for a Unique Raman Signature of Amyloid‐Beta Plaques in Human Brain Tissue From Alzheimer's Disease Patients,” Analyst 145, no. 5 (2020): 1724–1736. 31907497 10.1039/c9an02087j

[36] B. Lu , “BDNF and Activity‐Dependent Synaptic Modulation,” Learning & Memory 10, no. 2 (2003): 86–98. 12663747 10.1101/lm.54603 PMC5479144

[37] O. C. Küster , D. Laptinskaya , P. Fissler , et al., “Novel Blood‐Based Biomarkers of Cognition, Stress, and Physical or Cognitive Training in Older Adults at Risk of Dementia: Preliminary Evidence for a Role of BDNF, Irisin, and the Kynurenine Pathway,” Journal of Alzheimer's Disease 59, no. 3 (2017): 1097–1111. 10.3233/JAD-170447 28731451

[38] H. Tanila , “The Role of BDNF in Alzheimer's Disease,” Neurobiology of Disease 97 (2017): 114–118. 27185594 10.1016/j.nbd.2016.05.008

[39] F. Fumagalli , G. Racagni , and M. Riva , “The Expanding Role of BDNF: A Therapeutic Target for Alzheimer's Disease?,” Pharmacogenomics Journal 6, no. 1 (2006): 8–15. 16314887 10.1038/sj.tpj.6500337

[40] T. Numakawa and H. Odaka , “Brain‐Derived Neurotrophic Factor Signaling in the Pathophysiology of Alzheimer's Disease: Beneficial Effects of Flavonoids for Neuroprotection,” International Journal of Molecular Sciences 22, no. 11 (2021): 5719. 34071978 10.3390/ijms22115719 PMC8199014

[41] D. J. Garzon and M. Fahnestock , “Oligomeric Amyloid Decreases Basal Levels of Brain‐Derived Neurotrophic Factor (BDNF) mRNA via Specific Downregulation of BDNF Transcripts IV and V in Differentiated Human Neuroblastoma Cells,” Journal of Neuroscience 27, no. 10 (2007): 2628–2635. 17344400 10.1523/JNEUROSCI.5053-06.2007 PMC6672502

[42] Z. Zheng , B. Sabirzhanov , and J. Keifer , “Oligomeric Amyloid‐β Inhibits the Proteolytic Conversion of Brain‐Derived Neurotrophic Factor (BDNF), AMPA Receptor Trafficking, and Classical Conditioning,” Journal of Biological Chemistry 285, no. 45 (2010): 34708–34717. 20807770 10.1074/jbc.M110.150821 PMC2966086

[43] F. Zhang , Z. Kang , W. Li , Z. Xiao , and X. Zhou , “Roles of Brain‐Derived Neurotrophic Factor/Tropomyosin‐Related Kinase B (BDNF/TrkB) Signalling in Alzheimer's Disease,” Journal of Clinical Neuroscience 19, no. 7 (2012): 946–949. 22613489 10.1016/j.jocn.2011.12.022

[44] P. Girotra , T. Behl , A. Sehgal , S. Singh , and S. Bungau , “Investigation of the Molecular Role of Brain‐Derived Neurotrophic Factor in Alzheimer's Disease,” Journal of Molecular Neuroscience 72, no. 2 (2022): 173–186. 34424488 10.1007/s12031-021-01824-8

[45] C. Zuccato and E. Cattaneo , “Role of Brain‐Derived Neurotrophic Factor in Huntington's Disease,” Progress in Neurobiology 81, no. 5–6 (2007): 294–330. 17379385 10.1016/j.pneurobio.2007.01.003

[46] F. Kapczinski , B. N. Frey , A. C. Andreazza , M. Kauer‐Sant'Anna , Â. Cunha , and R. M. Post , “Increased Oxidative Stress as a Mechanism for Decreased BDNF Levels in Acute Manic Episodes,” Brazilian Journal of Psychiatry 30 (2008): 243–245. 18833425 10.1590/s1516-44462008000300011

[47] M. Calvo‐Rodriguez , S. S. Hou , A. C. Snyder , et al., “Increased Mitochondrial Calcium Levels Associated With Neuronal Death in a Mouse Model of Alzheimer's Disease,” Nature Communications 11, no. 1 (2020): 2146. 10.1038/s41467-020-16074-2 PMC7195480 32358564

[48] M. M. Anwar , “Oxidative Stress—A Direct Bridge to Central Nervous System Homeostatic Dysfunction and Alzheimer's Disease,” Cell Biochemistry and Function 40, no. 1 (2022): 17–27. 34716723 10.1002/cbf.3673

[49] A. Y. Abramov , L. Canevari , and M. R. Duchen , “Calcium Signals Induced by Amyloid β Peptide and Their Consequences in Neurons and Astrocytes in Culture. Biochimica et Biophysica Acta (BBA)‐Molecular,” Cell Research 1742, no. 1–3 (2004): 81–87. 10.1016/j.bbamcr.2004.09.006 15590058

[50] J. Zhao , G. Zhang , M. Li , Q. Luo , Y. Leng , and X. Liu , “Neuro‐Protective Effects of Aloperine in an Alzheimer's Disease Cellular Model,” Biomedicine & Pharmacotherapy 108 (2018): 137–143. 30218858 10.1016/j.biopha.2018.09.008

[51] S. Puoyan‐Majd , A. Parnow , M. Rashno , R. Heidarimoghadam , and A. Komaki , “The Protective Effects of High‐Intensity Interval Training Combined With Q10 Supplementation on Learning and Memory Impairments in Male Rats With Amyloid‐β‐Induced Alzheimer's Disease,” Journal of Alzheimer's Disease 99 (2023): S67–S80. 10.3233/JAD-230096 37212117

[52] F. G. M. Coelho , T. M. Vital , A. M. Stein , et al., “Acute Aerobic Exercise Increases Brain‐Derived Neurotrophic Factor Levels in Elderly With Alzheimer's Disease,” Journal of Alzheimer's Disease 39, no. 2 (2014): 401–408. 10.3233/JAD-131073 24164734

[53] C. Leger , A. Quirié , A. Méloux , et al., “Impact of Exercise Intensity on Cerebral BDNF Levels: Role of FNDC5/Irisin,” International Journal of Molecular Sciences 25, no. 2 (2024): 1213. 38279218 10.3390/ijms25021213 PMC10816613

[54] M. J. Chen , A. S. Ivy , and A. A. Russo‐Neustadt , “Nitric Oxide Synthesis Is Required for Exercise‐Induced Increases in Hippocampal BDNF and Phosphatidylinositol 3′ Kinase Expression,” Brain Research Bulletin 68, no. 4 (2006): 257–268. 16377431 10.1016/j.brainresbull.2005.08.013

[55] B. Xu , “BDNF (I) Rising From Exercise,” Cell Metabolism 18, no. 5 (2013): 612–614. 24206660 10.1016/j.cmet.2013.10.008 PMC3951409

[56] C.‐L. Tsai , C.‐Y. Pan , Y.‐T. Tseng , F.‐C. Chen , Y.‐C. Chang , and T.‐C. Wang , “Acute Effects of High‐Intensity Interval Training and Moderate‐Intensity Continuous Exercise on BDNF and Irisin Levels and Neurocognitive Performance in Late Middle‐Aged and Older Adults,” Behavioural Brain Research 413 (2021): 113472. 34274372 10.1016/j.bbr.2021.113472

[57] S. Camandola and M. P. Mattson , “Brain Metabolism in Health, Aging, and Neurodegeneration,” EMBO Journal 36, no. 11 (2017): 1474–1492. 28438892 10.15252/embj.201695810 PMC5452017

[58] E. Conti , D. Grana , G. Stefanoni , et al., “Irisin and BDNF Serum Levels and Behavioral Disturbances in Alzheimer's Disease,” Neurological Sciences 40 (2019): 1145–1150. 30810826 10.1007/s10072-019-03781-y

[59] R. Waseem , A. Shamsi , T. Mohammad , et al., “FNDC5/Irisin: Physiology and Pathophysiology,” Molecules 27, no. 3 (2022): 1118. 35164383 10.3390/molecules27031118 PMC8838669

[60] C. Wrann , J. White , J. Salogiannnis , et al., “Exercise Induces Hippocampal BDNF Through a PGC‐1alpha. FNDC5 Pathway,” Cell Metabolism 18 (2013): 649659. 10.1016/j.cmet.2013.09.008 PMC3980968 24120943

[61] A. Clin , “The Relationship Between High‐Fat Diet and Fibronectin Type‐III Domain‐Containing Protein 5 mRNA Expression,” Anatolian Clinic the Journal of Medical Sciences 23 (2018): 1–5.

[62] H.‐S. Moon , F. Dincer , and C. S. Mantzoros , “Pharmacological Concentrations of Irisin Increase Cell Proliferation Without Influencing Markers of Neurite Outgrowth and Synaptogenesis in Mouse H19‐7 Hippocampal Cell Lines,” Metabolism 62, no. 8 (2013): 1131–1136. 23664146 10.1016/j.metabol.2013.04.007 PMC4370428

[63] Y. Noda , A. Kuzuya , K. Tanigawa , et al., “Fibronectin Type III Domain‐Containing Protein 5 Interacts With APP and Decreases Amyloid β Production in Alzheimer's Disease,” Molecular Brain 11 (2018): 1–13. 30355327 10.1186/s13041-018-0401-8 PMC6201590

[64] L. S. Quinn , B. G. Anderson , J. D. Conner , and T. Wolden‐Hanson , “Circulating Irisin Levels and Muscle FNDC5 mRNA Expression Are Independent of IL‐15 Levels in Mice,” Endocrine 50 (2015): 368–377. 25920499 10.1007/s12020-015-0607-9

[65] O. Y. Kim and J. Song , “The Role of Irisin in Alzheimer's Disease,” Journal of Clinical Medicine 7, no. 11 (2018): 407. 30388754 10.3390/jcm7110407 PMC6262319

[66] S. Mohammadi , S. Oryan , A. Komaki , A. Eidi , and M. Zarei , “Effects of Intra‐Dentate Gyrus Microinjection of Myokine Irisin on Long‐Term Potentiation in Male Rats,” Arquivos de Neuro‐Psiquiatria 77 (2020): 881–887. 10.1590/0004-282X20190184 31939585

[67] M. E. Afzalpour , H. T. Chadorneshin , M. Foadoddini , and H. A. Eivari , “Comparing Interval and Continuous Exercise Training Regimens on Neurotrophic Factors in Rat Brain,” Physiology & Behavior 147 (2015): 78–83. 25868740 10.1016/j.physbeh.2015.04.012

[68] H. Shirvani and E. Arabzadeh , “Metabolic Cross‐Talk Between Skeletal Muscle and Adipose Tissue in High‐Intensity Interval Training vs. Moderate‐Intensity Continuous Training by Regulation of PGC‐1α,” Eating and Weight Disorders—Studies on Anorexia, Bulimia and Obesity 25 (2020): 17–24. 10.1007/s40519-018-0491-4 29480414

[69] E. Arabzadeh , H. Shirvani , M. Ebadi Zahmatkesh , S. Riyahi Malayeri , G. H. Meftahi , and F. Rostamkhani , “Irisin/FNDC5 Influences Myogenic Markers on Skeletal Muscle Following High and Moderate‐Intensity Exercise Training in STZ‐Diabetic Rats,” 3 Biotech 12, no. 9 (2022): 193. 10.1007/s13205-022-03253-9 PMC9325938 35910290

[70] I. Fatemi , P. S. Askari , E. Hakimizadeh , et al., “Chronic Treatment With Coenzyme Q10 Mitigates the Behavioral Dysfunction of Global Cerebral Ischemia/Reperfusion Injury in Rats,” Iranian Journal of Basic Medical Sciences 25, no. 1 (2022): 39–45. 35656440 10.22038/IJBMS.2022.57630.12865 PMC9118282

[71] M. Sheykhhasan , R. Amini , S. S. Asl , M. Saidijam , S. M. Hashemi , and R. Najafi , “Neuroprotective Effects of Coenzyme Q10‐Loaded Exosomes Obtained From Adipose‐Derived Stem Cells in a Rat Model of Alzheimer's Disease,” Biomedicine & Pharmacotherapy 152 (2022): 113224. 35679720 10.1016/j.biopha.2022.113224

[72] A. M. Youssef , D. A. Mohamed , S. Hussein , D. M. Abdullah , and S. A. Abdelrahman , “Effects of Quercetin and Coenzyme Q10 on Biochemical, Molecular, and Morphological Parameters of Skeletal Muscle in Trained Diabetic Rats,” Current Molecular Pharmacology 15, no. 1 (2022): 239–251. 34061009 10.2174/1874467214666210521170339

[73] X.‐L. Zhang , N. Zhao , B. Xu , X.‐H. Chen , and T.‐J. Li , “Treadmill Exercise Inhibits Amyloid‐β Generation in the Hippocampus of APP/PS1 Transgenic Mice by Reducing Cholesterol‐Mediated Lipid Raft Formation,” NeuroReport 30, no. 7 (2019): 498–503. 30882716 10.1097/WNR.0000000000001230

[74] X.‐f. He , D.‐x. Liu , Q. Zhang , et al., “Voluntary Exercise Promotes Glymphatic Clearance of Amyloid Beta and Reduces the Activation of Astrocytes and Microglia in Aged Mice,” Frontiers in Molecular Neuroscience 10 (2017): 144. 28579942 10.3389/fnmol.2017.00144 PMC5437122

[75] P. A. Adlard , V. M. Perreau , V. Pop , and C. W. Cotman , “Voluntary Exercise Decreases Amyloid Load in a Transgenic Model of Alzheimer's Disease,” Journal of Neuroscience 25, no. 17 (2005): 4217–4221. 15858047 10.1523/JNEUROSCI.0496-05.2005 PMC6725122

[76] Z. Radak , N. Hart , L. Sarga , et al., “Exercise Plays a Preventive Role Against Alzheimer's Disease,” Journal of Alzheimer's Disease 20, no. 3 (2010): 777–783. 10.3233/JAD-2010-091531 20182027

[77] R. Thomas , S. D. Zimmerman , K. M. Yuede , et al., “Exercise Training Results in Lower Amyloid Plaque Load and Greater Cognitive Function in an Intensity Dependent Manner in the tg2576 Mouse Model of Alzheimer's Disease,” Brain Sciences 10, no. 2 (2020): 88. 32046299 10.3390/brainsci10020088 PMC7071605

[78] D. Khodadadi , R. Gharakhanlou , N. Naghdi , et al., “Treadmill Exercise Ameliorates Spatial Learning and Memory Deficits Through Improving the Clearance of Peripheral and Central Amyloid‐Beta Levels,” Neurochemical Research 43 (2018): 1561–1574. 29948724 10.1007/s11064-018-2571-2

[79] H.‐S. Um , E.‐B. Kang , J.‐H. Koo , et al., “Treadmill Exercise Represses Neuronal Cell Death in an Aged Transgenic Mouse Model of Alzheimer's Disease,” Neuroscience Research 69, no. 2 (2011): 161–173. 20969897 10.1016/j.neures.2010.10.004

[80] H.‐l. Liu , G. Zhao , and H. Zhang , “Long‐Term Treadmill Exercise Inhibits the Progression of Alzheimer's Disease‐Like Neuropathology in the Hippocampus of APP/PS1 Transgenic Mice,” Behavioural Brain Research 256 (2013): 261–272. 23968591 10.1016/j.bbr.2013.08.008

[81] H. S. Um , E. B. Kang , Y. H. Leem , et al., “Exercise Training Acts as a Therapeutic Strategy for Reduction of the Pathogenic Phenotypes for Alzheimer's Disease in an NSE/APPsw‐Transgenic Model,” International Journal of Molecular Medicine 22, no. 4 (2008): 529–539. 18813861

[82] P. Pignataro , M. Dicarlo , R. Zerlotin , et al., “FNDC5/Irisin System in Neuroinflammation and Neurodegenerative Diseases: Update and Novel Perspective,” International Journal of Molecular Sciences 22, no. 4 (2021): 1605. 33562601 10.3390/ijms22041605 PMC7915567

[83] C. Elipenahli , C. Stack , S. Jainuddin , et al., “Behavioral Improvement After Chronic Administration of Coenzyme Q10 in P301S Transgenic Mice,” Journal of Alzheimer's Disease 28, no. 1 (2012): 173–182. 10.3233/JAD-2011-111190 PMC3279177 21971408

[84] X. Yang , G. Dai , G. Li , and E. S. Yang , “Coenzyme Q10 Reduces β‐Amyloid Plaque in an APP/PS1 Transgenic Mouse Model of Alzheimer's Disease,” Journal of Molecular Neuroscience 41 (2010): 110–113. 19834824 10.1007/s12031-009-9297-1

[85] M. Dumont , K. Kipiani , F. Yu , et al., “Coenzyme Q10 Decreases Amyloid Pathology and Improves Behavior in a Transgenic Mouse Model of Alzheimer's Disease,” Journal of Alzheimer's Disease 27, no. 1 (2011): 211–223. 10.3233/JAD-2011-110209 PMC3267988 21799249

[86] X. Yang , Y. Yang , G. Li , J. Wang , and E. S. Yang , “Coenzyme Q10 Attenuates β‐Amyloid Pathology in the Aged Transgenic Mice With Alzheimer Presenilin 1 Mutation,” Journal of Molecular Neuroscience 34 (2008): 165–171. 18181031 10.1007/s12031-007-9033-7

